# Screening and Response for Adverse Social Determinants of Health in US Emergency Departments

**DOI:** 10.1001/jamanetworkopen.2025.7951

**Published:** 2025-04-23

**Authors:** Melanie F. Molina, Rebecca E. Cash, Stephanie S. Loo, Maeve F. Swanton, Janice A. Espinola, Krislyn M. Boggs, Olivia Chen, Alan J. Ardelean, Carlos A. Camargo, Margaret E. Samuels-Kalow

**Affiliations:** 1 Department of Emergency Medicine, University of California, San Francisco; 2 Philip R. Lee Institute for Health Policy Studies, University of California, San Francisco; 3 Department of Emergency Medicine, Massachusetts General Hospital, Harvard Medical School, Boston; 4 Department of Emergency Medicine, University of Massachusetts Medical School, Worcester; 5 Department of Quantitative Health Sciences, University of Massachusetts Medical School, Worcester

## Abstract

**Importance:**

Regulatory agencies have begun incentivizing screening for adverse social determinants of health (SDOH) and responses in inpatient settings, missing a crucial safety net: the emergency department (ED). Little is known about the prevalence of ED-based adverse SDOH screening and response practices nationally.

**Objective:**

To describe the prevalence of ED-based adverse SDOH screening and response policies and to identify associated hospital characteristics.

**Design, Setting, and Participants:**

This survey study utilized a 5% random sample from the National Emergency Department Inventory–USA, including EDs stratified by geography, urbanicity, and practice setting (academic vs community). Data regarding 2022 policies were collected in 2023.

**Exposures:**

Practice setting, urbanicity, visit volume, and availability of social work.

**Main Outcomes and Measures:**

The presence of written policies for any adverse SDOH (housing, food, transportation, and utility payment difficulties) screening and responses, as well as other requirement-driven screening for SDOH risk factors (intimate partner violence, substance use, and mental health conditions). Responses were categorized as consultations (eg, social work), standardized information sheets, individualized resource information, or other.

**Results:**

Of a total of 280 EDs, 232 responded (83% response rate). Among 232 EDs, 28.4% (survey-weighted proportion; 95% CI, 21.0%-37.2%) had screening policies for at least 1 adverse SDOH domain, and 93.1% (95% CI, 89.2%-95.7%) performed at least 1 other requirement-driven screening (eg, intimate partner violence). Of EDs performing any screening (adverse SDOH or other), 81.6% (95% CI, 73.4%-87.7%) had response policies, primarily involving consultations (78.2%; 95% CI, 67.2%-86.3%), standardized information sheets (43.0%; 95% CI, 32.5%-54.3%), and individualized resource information (12.9%; 95% CI, 7.2%-21.8%). Among all responding EDs, only 23.4% (95% CI, 17.1%-31.2%) had around-the-clock social work availability, and 20.5% (95% CI, 14.2%-28.6%) had an ED-based social worker. There was no association between practice setting, urbanicity, visit volume, or around-the-clock social work with adverse SDOH screening or response policies.

**Conclusions and Relevance:**

Despite the high prevalence of adverse SDOH in ED populations, in this survey study of 232 EDs, less than one-third performed screening, and one-fifth did not have policies requiring a response to positive screens. Bridging this gap may require expanding adverse SDOH screening practices while also ensuring that EDs have the resources and infrastructure to respond appropriately to identified social needs. Future research might explore advanced technological solutions to enhance screening and responses in these resource-constrained settings.

## Key Points

**Question:**

What is the prevalence of adverse social determinants of health (SDOH) screening and response policies in US emergency departments (EDs)?

**Findings:**

In this national cross-sectional survey of 232 EDs, only 28.4% screened for adverse SDOH. Among EDs with screening policies, 81.6% had response policies in place, primarily utilizing social work consultations, although only 20.5% had an ED-based social worker and 23.4% had around-the-clock social work availability.

**Meaning:**

These findings highlight a substantial gap in the screening and responses for adverse SDOH in US EDs, underscoring the need for enhanced resources and infrastructure to better address social needs in vulnerable ED populations.

## Teaser

This survey study examines the prevalence of emergency department (ED)–based screening for adverse social determinants of health and response policies and identifies associated hospital characteristics.

## Introduction

Adverse social determinants of health (SDOH), such as food insecurity or housing instability, are associated with poor health outcomes and increased health care utilization.^1,2,3,4^ Recognizing the importance of identifying adverse SDOH, regulatory agencies such as the Centers for Medicare & Medicaid Services and The Joint Commission have begun incentivizing health care systems to perform multidomain adverse SDOH screening.^5^ However, less emphasis has been placed on adverse SDOH–related responses, which are equally important as screening, if not more so. Furthermore, these efforts have focused primarily on inpatient hospitalizations and outpatient settings,^5^ missing a crucial safety net: the emergency department (ED).

The ED provides an opportunity to screen hard-to-reach, vulnerable populations whose adverse SDOH may not be identified in other settings.^6,7,8^ Approximately 25% of adult Americans do not have access to primary care,^9^ and many resort to the ED for routine care.^10^ Furthermore, ED populations have a high prevalence of adverse SDOH,^11^ with one study reporting more than one-third of ED patients screening positive for 1 or more adverse SDOH risk factors.^7^ Nevertheless, most adverse SDOH screening tools and responses have been studied in outpatient settings,^12,13,14^ with tools like the Protocol for Responding to and Assessing Patients’ Assets, Risks, and Experiences and the Accountable Health Communities screening often having multiple questions to assess a single adverse SDOH.^15,16^ This makes them difficult to adapt to the fast-paced, resource-constrained ED environment.^17^ Exploring how these adverse SDOH care-related activities can be tailored and implemented in EDs is an area of active research.^18^

Currently, little is known about the national prevalence of ED-based adverse SDOH screening and response practices, and a wide variation has been described in the literature.^6,18^ One study examining 166 EDs in New England found that 39% reported screening for at least 1 adverse SDOH in 2018.^6^ A lack of validated ED-based screening tools contributes to inconsistent screening across specific domains,^18^ and responses vary from immediate meal provision to resource handouts.^19^ Illuminating the national landscape of adverse SDOH screening and response practices is necessary to identify areas where improvement is needed. Furthermore, an analysis of the variation in these practices can provide important clues as to how different EDs are adapting to the unique challenges and needs of their patient populations—helping inform subsequent best practices for optimizing the integration of social care into emergency medicine. Therefore, we sought to describe the prevalence of ED screening and response policies for adverse SDOH in a nationally representative sample and to identify hospital characteristics (eg, urban or rural, academic or community) associated with the implementation of screening and response policies.

## Methods

### Design, Setting, and Population

We conducted a cross-sectional survey study of a 5% stratified random sample from the National Emergency Department Inventory (NEDI-USA). The NEDI-USA database contains survey data from over 5500 nonfederal EDs across the US and is collected annually by the Emergency Medicine Network^20^; its methods have been previously reported.^21^ Questions related to screening for adverse SDOH were first investigated in the NEDI-New England study in 2018.^6^

Building on the 2018 survey, we targeted the current survey to EDs throughout the US. Therefore, on the basis of the most recently available NEDI-USA data (2020 NEDI-USA), we randomly sampled 280 (approximately 5%) EDs, stratifying by geographic region, urbanicity, and practice setting (ie, academic vs community) to ensure diversity. We excluded freestanding EDs.^22^ The Mass General Brigham Research Committee classified this work as exempt from review and the need for consent in accordance with institutional policy related to human participants research. We followed the Strengthening the Reporting of Observational Studies in Epidemiology (STROBE) reporting guidelines.

### Survey Development and Administration

On the basis of input from physicians and health services researchers, we developed questions regarding ED-based adverse SDOH screening policies and responses (eAppendix in Supplement 1). We inquired about written screening policies spanning multiple domains (ie, housing instability, food insecurity, transportation difficulties, utilities, intimate partner violence, other exposure to violence, substance use, and mental health), written response policies in response to positive screens, and the availability of social work services. Within each domain, if screening was reported, ED leaders were subsequently asked whether there were written policies requiring a specific response in response to positive screens. These responses were categorized as a consultation (eg, social work), the provision of standardized information, the provision of individualized information (eg, using UniteUs or other individual resource mapping software), and a free-text option (ie, other). Leaders were allowed to check all categories that applied. Emergency Medicine Network staff mailed the modified surveys to the primary point of contact for each ED up to 3 times over a 2-month period. Respondents were primarily ED or medical directors. The surveys could either be completed online or returned by mail. Nonresponding EDs were contacted by phone to complete the survey via interview. We collected data from January to October 2023 and asked about policies pertaining to the year 2022.

### Definitions, Outcomes, and Measures

Our primary outcomes were the presence or absence of adverse SDOH screening and response policies. EDs had a positive adverse SDOH screening status if they had a written policy to screen for any of the following: housing instability, food insecurity, transportation difficulties, and trouble paying for utilities. Response status was defined similarly but was only ascertained among those EDs that reported screening. The adverse SDOH screening domains were based on consensus screening recommendations for health-related social needs in clinical settings.^23^ Other screening domains included intimate partner violence, other exposure to violence, substance use, and mental health conditions (eg, suicide). These risk factors were designated as other requirement-driven screening rather than adverse SDOH screening status because they have different screening recommendations (ie, mandated by regulatory agencies) and are therefore commonly screened for in the ED. We examined associations between the primary outcomes and key ED characteristics, including visit volumes, urbanicity (based on location in a core-based statistical area^21^), practice setting (academic vs community, defined by membership in the Council of Teaching Hospitals), and the availability of social work services.

### Statistical Analysis

We summarized and compared ED characteristics by adverse SDOH screening status using survey-weighted proportions with 95% CIs and Taylor series linearization for variance estimation. Survey weights were created on the basis of the complex sampling design using iterative proportional fitting (ie, raking). Raking is an iterative process that calibrates poststratification weights to match target population control totals according to auxiliary information. Starting weights to represent the overall 2022 NEDI-USA population were calculated by the inverse probability of response in each of the sampling strata (geographic region, urbanicity, and practice setting). Population totals for these variables were determined, and raking was used to adjust initial weights.^24^ The final survey weights, after raking, adjusted the expected proportions of certain variables to match the proportions found in the target ED population.

For EDs that had screening policies, we examined the presence of response policies by screening domain and further categorized responses among EDs reporting the presence of at least 1 response policy for any social risk domain. We explored the association of ED characteristics with both adverse SDOH screening and responses, each as a binary outcome, using survey-weighted multivariable logistic regression models to produce odds ratios (ORs) with 95% CIs. Statistical significance was defined as a 95% CI that did not include the null value of 1. All statistical analyses were specified a priori on the basis of factors we hypothesized to be associated with adverse SDOH screening and response policies and were performed using Stata statistical software version 18.0 (StataCorp).

## Results

### ED Characteristics

Of the 280 EDs surveyed, 232 (83%) responded to the screening policy questions, representing approximately 4% of the 5622 NEDI-USA EDs in 2022. Among responding EDs, the weighted prevalence of academic EDs was 4.6% (95% CI, 4.5%-4.7%), 11.3% (95% CI, 11.1%-11.5) were in the Northeast, 26.4% (95% CI, 25.2%-27.7%) were in the Midwest, 43.6% (95% CI, 42.8%-44.3%) were in the South, 18.7% (95% CI, 18.4%-19.0%) were in the West, 80.5% (95% CI, 80.1%-80.8%) were urban, and the mean total ED yearly visits was 28 327 (95% CI, 24 845-31 810 visits). Although 92.2% (95% CI, 86.9%-95.5%) of EDs had social work services available, only 20.5% (95% CI, 14.2%-28.6%) had an ED-based social worker, and 23.4% (95% CI, 17.1%-31.2%) had social work services available 24 hours per day, 7 days per week (24/7) (Table 1).

**Table 1.** ED Characteristics by Adverse Social Determinants of Health Screening Status (Excluding Requirement-Driven Screening)

Characteristics	All EDs (N = 232)		No screening (n = 155)		Screening (n = 77)	
	Unweighted No.	Weighted % (95% CI)^a^	Unweighted No.	Weighted % (95% CI)^a^	Unweighted No.	Weighted % (95% CI)^a^
Academic						
No	166	95.4 (95.3-95.5)	119	96.3 (95.4-97.1)	47	93.0 (89.7-95.3)
Yes	66	4.6 (4.5-4.7)	36	3.7 (2.9-4.6)	30	7.0 (4.7-10.3)
Region						
Northeast	58	11.3 (11.1-11.5)	36	10.6 (8.4-13.2)	22	13.1 (8.0-20.7)
Midwest	60	26.4 (25.2-27.7)	42	26.5 (21.9-31.7)	18	26.1 (15.9-39.7)
South	60	43.6 (42.8-44.3)	45	47.1 (41.4-52.9)	15	34.6 (20.9-51.4)
West	54	18.7 (18.4-19.0)	32	15.8 (12.1-20.3)	22	26.2 (16.9-38.2)
Urbanicity						
Rural	73	19.5 (19.2-19.9)	55	20.9 (17.8-24.4)	18	16.1 (9.8-25.4)
Urban	159	80.5 (80.1-80.8)	100	79.1 (75.6-82.2)	59	83.9 (74.6-90.2)
Total ED visits, mean (95% CI)	232	28 327 (24 845-31 810)	155	26 295 (22 214-30 377)	77	33 451 (26 408-40 493)
Total ED visits						
<10 000	67	21.9 (18.2-26.0)	48	22.1 (17.2-28.0)	19	21.2 (12.6-33.4)
10 000-19 999	34	21.6 (14.7-30.7)	29	25.5 (16.9-36.6)	5	11.8 (3.9-30.7)
20 000-39 999	47	36.7 (28.3-46.0)	32	33.7 (23.9-45.3)	15	44.2 (28.7-60.9)
≥40 000	84	19.8 (13.8-27.5)	46	18.6 (11.4-28.8)	38	22.8 (14.0-35.1)
Child ED visits, mean (95% CI)	227	5176 (3538-6813)	150	5312 (3106-7519)	77	4839 (3293-6386)
At least 1 attending physician on duty in the ED at all times						
No	23	7.7 (5.0-11.6)	19	8.3 (5.2-12.9)	4	6.3 (2.0-17.9)
Yes	208	92.3 (88.4-95.0)	135	91.7 (87.1-94.8)	73	93.7 (82.1-98.0)
Dedicated area for children						
No	171	79.8 (71.3-86.3)	117	80.0 (69.3-87.6)	54	79.6 (63.3-89.8)
Yes	54	16.8 (11.0-24.9)	32	15.5 (9.0-25.4)	22	20.1 (9.9-36.4)
Not applicable (eg, children’s hospital)	7	3.3 (1.1-9.7)	6	4.5 (1.4-13.3)	1	0.3 (0.0-2.4)
Availability of social work services in ED						
ED-based social worker(s)	72	20.5 (14.2-28.6)	38	16.0 (9.6-25.6)	34	31.7 (18.9-48.1)
Hospital social worker(s) based outside the ED but can respond to the ED	86	49.8 (40.6-58.9)	64	52.6 (41.6-63.3)	22	42.7 (27.3-59.7)
Mixture of ED-based and hospital social worker(s)	40	18.3 (12.0-26.9)	26	18.4 (11.0-29.1)	14	18.1 (8.5-34.6)
Other	12	3.7 (2.0-6.7)	10	4.4 (2.2-8.5)	2	2.0 (0.5-8.1)
None	22	7.8 (4.5-13.1)	17	8.7 (4.7-15.6)	5	5.5 (1.6-17.0)
Availability of social work services in ED						
None	22	7.8 (4.5-13.1)	17	8.7 (4.7-15.6)	5	5.5 (1.6-17.0)
Any	210	92.2 (86.9-95.5)	138	91.3 (84.4-95.3)	72	94.5 (83.0-98.4)
Social work services available in ED at all times						
No	151	76.6 (68.8-82.9)	110	80.4 (71.0-87.2)	41	67.0 (51.0-79.9)
Yes	81	23.4 (17.1-31.2)	45	19.6 (12.8-29.0)	36	33.0 (20.1-49.0)

Abbreviation: ED, emergency department.

^a^ Percentages are survey-weighted based on the 2022 National Emergency Department Inventory–USA population, representing 5622 nonfederal EDs in the US.

### Screening Policies for Adverse SDOH

The weighted prevalence of EDs that reported having written policies to screen for at least 1 adverse SDOH (housing instability, food insecurity, transportation difficulties, or trouble paying for utilities) was 28.4% (95% CI, 21.0%-37.2%), whereas 71.6% (95% CI, 62.8%-79.0%) did not perform adverse SDOH screening. In contrast, 93.1% (95% CI, 89.2%-95.7%) of EDs reported screening for at least 1 other requirement-driven risk factor, including intimate partner violence, other exposure to violence, substance use, and mental health conditions (eg, suicide or psychosis). Stratifying by adverse SDOH screening status, EDs that screened for at least 1 adverse SDOH were more often academic and tended toward higher average ED volumes. For each individual adverse SDOH domain, 22.7% (95% CI 16.3%-30.6%) of EDs had policies to screen for housing instability, 14.9% (95% CI 9.6%-22.5%) for food insecurity, 13.1% (95% CI, 8.2%-20.2%) for difficulty obtaining transportation, and 4.0% (95% CI, 2.1%-7.6%) for trouble paying for utilities. For the other requirement-driven screening domains, 80.0% (95% CI, 72.4%-86.0%) of EDs reported having policies to screen for intimate partner violence, 71.8% (95% CI, 63.6%-78.7%) for other exposure to violence, 81.2% (95% CI, 74.4%-86.6%) for substance use, and 90.3% (95% CI, 84.9%-94.0%) for mental health conditions (Table 2).

**Table 2.** Prevalence of Adverse Social Determinants of Health Screening in US Emergency Departments

Screening domains	Unweighted No. (n = 232)	Weighted % (95% CI)
Individual		
Housing instability	64	22.7 (16.3-30.6)
Food insecurity	45	14.9 (9.6-22.5)
Transportation difficulties	41	13.1 (8.2-20.2)
Trouble paying utilities	14	4.0 (2.1-7.6)
Intimate partner violence	179	80.0 (72.4-86.0)
Other exposure to violence	146	71.8 (63.6-78.7)
Substance use	170	81.2 (74.4-86.6)
Mental health	204	90.3 (84.9-94.0)
Composite		
Any adverse social determinant of health^a^	77	28.4 (21.0-37.2)
Other requirement-driven screening^b^	212	93.1 (89.2-95.7)

^a^ Refers to housing instability, food insecurity, transportation difficulties, and trouble paying for utilities.

^b^ Refers to intimate partner violence, other exposure to violence, substance use, or mental health conditions.

### Response Policies for Adverse SDOH

Of all EDs that reported having a policy to screen for at least 1 adverse SDOH or other requirement-driven risk factor, 81.6% (95% CI, 73.4%-87.7%) reported having policies requiring some type of response. Of the EDs that reported having policies requiring responses for any social need, 78.2% (95% CI, 67.2%-86.3%) reported using consultation services (ie, social work), 43.0% (95% CI, 32.5%-54.3%) provided standardized information sheets, 12.9% (95% CI, 7.2%-21.8%) provided individualized information (eg, UniteUs or other individual resource mapping software), and 10.8% (95% CI, 5.4%-20.3%) reported delivering some type of other response. When we examined the types of responses that were reported when EDs had at least 1 response policy for any social risk domain, we found that consultation was a listed response in 70% to 100% of response policies across all domains, particularly for those related to housing instability (84.8%; 95% CI, 61.7%-95.1%), transportation difficulties (96.0%; 95% CI, 80.9%-99.3%), and trouble paying for utilities (100.0%). The provision of standardized information sheets was also listed as a common response when EDs reported having response policies for housing instability (51.8%; 95% CI, 33.0%-70.1%) and trouble paying for utilities (57.6%; 95% CI, 23.8%-85.5%), whereas the provision of individualized information much was less common (<25%) across all social risk domains except EDs reporting a response policy for trouble paying for utilities (39.3%; 95% CI, 13.3%-73.1%) (Table 3).

**Table 3.** Presence of Policies Requiring a Response to Unmet Needs Among Emergency Departments With Screening Policies, by Need

Policy requiring a specified response	Weighted % (95% CI)								
	Any unmet need (n = 168)^a^	Housing instability	Food insecurity	Transportation difficulties	Trouble paying utilities	Intimate partner violence	Other exposure to violence	Substance use	Mental health
Consultation									
No	21.8 (13.7-32.8)	15.2 (4.9-38.3)	27.4 (8.8-59.7)	4.0 (0.7-19.1)	NA	19.8 (11.7-31.5)	22.5 (13.5-35.1)	21.8 (13.3-33.6)	21.8 (13.7-32.8)
Yes	78.2 (67.2-86.3)	84.8 (61.7-95.1)	72.6 (40.3-91.2)	96.0 (80.9-99.3)	100.0	80.2 (68.5-88.3)	77.5 (64.9-86.5)	78.2 (66.4-86.7)	78.2 (67.2-86.3)
Standardized information sheets									
No	57.0 (45.7-67.5)	48.2 (29.9-67.0)	66.5 (39.6-85.7)	81.2 (59.6-92.6)	42.4 (14.5-76.2)	57.0 (45.2-68.0)	54.0 (41.7-65.8)	53.3 (41.6-64.7)	56.9 (45.7-67.5)
Yes	43.0 (32.5-54.3)	51.8 (33.0-70.1)	33.6 (14.3-60.4)	18.8 (7.4-40.4)	57.6 (23.8-85.5)	43.0 (32.0-54.8)	46.0 (34.2-58.3)	46.7 (35.4-58.4)	43.1 (32.5-54.3)
Individualized information									
No	87.2 (78.2-92.8)	77.6 (59.7-89.1)	78.9 (55.4-91.9)	75.8 (50.0-90.7)	60.7 (26.9-86.7)	85.9 (76.0-92.1)	85.0 (74.4-91.7)	85.8 (75.9-92.0)	87.1 (78.2-92.8)
Yes	12.9 (7.2-21.8)	22.4 (10.9-40.3)	21.1 (8.1-44.6)	24.2 (9.3-50.0)	39.3 (13.3-73.1)	14.1 (7.9-24.0)	15.0 (8.3-25.6)	14.3 (8.0-24.1)	12.9 (7.3-21.8)
Other response									
No	89.2 (79.7-94.)6	99.7 (97.8-100.0)	87.2 (48.1-98.0)	95.4 (81.4-99.0)	100.0	89.9 (79.5-95.3)	92.8 (82.2-97.3)	92.2 (82.9-96.6)	89.2 (79.7-94.6)
Yes	10.8 (5.4-20.3)	0.3 (0.0-2.2)	12.9 (2.0-51.9)	4.6 (1.0-18.6)	NA	10.1 (4.7-20.5)	7.2 (2.7-17.8)	7.8 (3.4-17.1)	10.8 (5.4-20.3)

Abbreviation: NA, not applicable.

^a^ Refers to housing instability, food insecurity, transportation difficulties, trouble paying for utilities, intimate partner violence, other exposure to violence, substance use, or mental health conditions.

### Characteristics Associated With Adverse SDOH Screening and Responses

We used weighted multivariable logistic regression to explore whether specific ED characteristics were associated with either policies to screen for or respond to any adverse SDOH. In a model adjusting for practice setting (academic vs community), urbanicity, and visit volume, we did not find a statistically significant association between 24/7 availability of social work services and any adverse SDOH screening policy (OR, 1.90; 95% CI, 0.79-4.58) (Table 4), as well as 24/7 availability of social work services and any adverse SDOH response policy (OR, 1.68; 95% CI, 0.58-4.88) (Table 5).

**Table 4.** Association of ED Characteristics With Adverse Social Determinants of Health Screening Status

Characteristics	OR (95% CI)^a^
Academic	1.40 (0.54-3.59)
Urban	1.99 (0.47-8.47)
Total ED visits	
<10 000	1 [Reference]
10 000-19 999	0.31 (0.05-1.79)
20 000-39 999	0.79 (0.16-3.90)
≥40 000	0.63 (0.12-3.32)
Social work services available in ED at all times	1.90 (0.79-4.58)

Abbreviations: ED, emergency department; OR, odds ratio.

^a^ ORs were calculated from survey-weighted logistic regression model.

**Table 5.** Association of ED Characteristics With Response for Any Adverse Social Determinants of Health Among EDs With Screening Policies

Characteristics	OR (95% CI)^a^
Academic	1.09 (0.33-3.57)
Urban	1.50 (0.33-6.83)
Total ED visits	
<10 000	1 [Reference]
10 000-19 999	0.60 (0.09-3.87)
20 000-39 999	1.12 (0.20-6.31)
≥40 000	0.83 (0.13-5.21)
Social work services available in ED at all times	1.68 (0.58-4.88)

Abbreviations: ED, emergency department; OR, odds ratio.

^a^ ORs were calculated from survey-weighted logistic regression model.

## Discussion

In this cross-sectional survey study of 232 US EDs in 2022, we found that less than one-third of EDs reported screening for at least 1 adverse SDOH (ie, housing instability, food insecurity, transportation difficulties, trouble paying for utilities); however, more than 90% of EDs reported screening for requirement-driven risk factors, including intimate partner violence, substance use, and mental health conditions. Although the vast majority of EDs had some social work services available, only 23.4% had 24/7 social work coverage, and even fewer (20.5%) had an ED-based social worker. Among EDs with screening policies for adverse SDOH or other requirement-driven risk factors, approximately 80% had policies requiring responses for positive screens, with the most common response being consultation services and standardized information sheets. We did not find any significant associations between practice setting, urbanicity, visit volume, and 24/7 availability of social work services with either adverse SDOH screening or response policies.

To our knowledge, this is the first study to estimate the national prevalence of adverse SDOH screening and response policies in US EDs. These results are consistent with our prior work in New England EDs, which found a slightly higher prevalence of screening policies for any adverse SDOH at 39%, including several individual domains, such as housing (33%), transportation (17%), and utilities (6%).^6^ The regional study also reflected similar trends toward screening more frequently for violence, substance use, and mental health conditions than for adverse SDOH.^6^ The slightly lower overall screening proportions in our current study reflect a broader variation in practices across different US regions, with the Northeast and West regions of the US tending toward relatively more screening than the Midwest and South regions (Table 1).

The discrepancy between the high prevalence of adverse SDOH among vulnerable ED populations^7,11,25,26,27^ and the low rates of screening impedes the delivery of high-quality, comprehensive care that both considers and addresses unmet social needs in clinical care plans. It risks perpetuating poor health outcomes and exacerbating existing health disparities. Given the potential impact of identifying and addressing adverse SDOH in ED populations, incorporating ED-based adverse SDOH screening into the mandates of regulatory bodies like Centers for Medicare & Medicaid Services and The Joint Commission could enhance screening rates nationwide. However, such mandates must also consider ED-specific time and resource constraints to avoid further burdening an already stressed system. Despite research indicating that both patients and physicians view ED-based adverse SDOH screening positively,^28,29^ cited barriers to screening include lack of knowledge, time constraints, and high patient volumes.^28^ Some investigators have questioned the need for screening altogether, highlighting its potential to propagate bias and stigma, and instead propose providing a resource menu for patients to self-select programs and services of interest.^30^ Another approach could involve financially incentivizing social responses through ED billing codes to increase the Relative Value Units of specific encounters. The integration of advanced technologies such as large language models or ambient scribe technology into screening processes, when combined with automated resource referrals, could represent yet another promising opportunity to bypass resource-intensive screening and linkage processes, thus reducing the burden on human resources. We encourage further research into these specific technological use cases.

Another crucial screening consideration is that the effectiveness and ethics of adverse SDOH screening depend entirely on the ability of EDs to respond to positive screens with the appropriate responses. Despite this, approximately 20% of EDs in our sample did not report having policies requiring a response to positive screens. Screening without response not only represents wasted resources but also risks inducing trauma and eroding patient trust and confidence in the health care system to address their needs.^31,32,33^ Nevertheless, it is important to note that the ability of EDs to respond to positive screens also depends on the availability of community resources, which has been cited as a key barrier to resource linkage.^34,35^ Therefore, policies that mandate or incentivize health care systems to perform adverse SDOH screening should include responses, but also ensure sufficient investment in community resource infrastructure.

Approximately 75% of EDs in our sample that required responses reported using consultation services (eg, social work) to address positive screens. However, fewer than 1 in 4 EDs had ED-based social work services or offered these services 24/7. Furthermore, most housing, employment, and financial services that might be used by social workers to address adverse SDOH are typically available during business hours only. Given that EDs operate 24/7, these findings raise questions both about the feasibility of providing social care responses outside regular business hours and the potential increased responsibility placed on ED staff and physicians to address adverse SDOH when social work consultation is unavailable. Beyond limiting resource linkage and care coordination, reduced social work availability delays discharge planning, prolongs ED stay, and can lead to unnecessary admissions, contributing to increased health care costs.^36^ Creative solutions such as telehealth services, financially incentivizing EDs to expand their social work services, or technological innovations such as automated, closed-loop referrals, may be necessary to provide around-the-clock support for patients experiencing adverse SDOH. Next steps include working to bridge the gap between identifying adverse SDOH and delivering timely responses in ED settings without overburdening staff and physicians.

### Limitations

Our study had several limitations, most of which were related to the nature of survey data collection. First, because our questions were concise and close-ended, we were not able to investigate details regarding the impetus behind screening, what questions were asked, how screening was performed, how frequently it was performed, or whether it was targeted or universal. We are actively exploring these factors in a separate qualitative study. Second, the self-reported survey responses may have been subject to elements of social desirability bias, which would likely bias our findings toward higher adverse SDOH screening and response rates. This would lead to an overestimated prevalence of screening and responses, further magnifying the importance of our findings. Although we did not find any statistically significant associations between ED characteristics and either adverse SDOH screening or response policies, our sample size may not have been sufficiently powered; larger national studies might better elucidate any associations. Third, our questions did not distinguish between screening for adverse SDOH (social risk) vs social need (social risk for which assistance is desired). These important, yet distinct concepts can be discordant but are often overlapping.^7,37,38,39^ Fourth, we acknowledge that written policies to screen for adverse SDOH do not always necessarily translate into screening practice. This suggests the need to evaluate fidelity to written policies for adverse SDOH where they do exist.

## Conclusions

In this survey study of 232 EDs, although most US EDs reported having policies to screen for behavioral determinants of health, such as substance use and mental health conditions, only about one-quarter of EDs screened for adverse SDOH. The most common response to positive screens was consultation (eg, social work), yet the vast majority of EDs did not have consistent, 24/7 social work availability. These findings represent a missed opportunity to reach and address the needs of vulnerable ED populations experiencing high rates of adverse SDOH. Bridging this gap may require not only expanding adverse SDOH screening practices but also ensuring that EDs have the resources and infrastructure in place to respond appropriately to identified social needs. Future research might explore advanced technological solutions to enhance screening and response processes in these resource-constrained settings.

## Notes

Supplement 1. **eAppendix.** Questions Regarding Emergency Department–Based Adverse Social Determinants of Health Screening Policies

Supplement 2.
Data Sharing Statement

## References

[1] Berkowitz SA, Baggett TP, Edwards ST. Addressing health-related social needs: value-based care or values-based care? J Gen Intern Med. 2019;34(9):1916-1918. doi:10.1007/s11606-019-05087-3 31183686 PMC6712198

[2] Samuel LJ, Szanton SL, Cahill R, . Does the Supplemental Nutrition Assistance Program affect hospital utilization among older adults? the case of Maryland. Popul Health Manag. 2018;21(2):88-95. doi:10.1089/pop.2017.0055 28683219 PMC5906726

[3] Harris DA, Mainardi A, Iyamu O, . Improving the asthma disparity gap with legal advocacy? a qualitative study of patient-identified challenges to improve social and environmental factors that contribute to poorly controlled asthma. J Asthma. 2018;55(8):924-932. doi:10.1080/02770903.2017.1373393 28872933

[4] Spencer A, Freda B, McGinnis T, Gottlieb L. Measuring social determinants of health among Medicaid beneficiaries: early state lessons. Center for Health Care Strategies, Inc. December 2016. Accessed March 14, 2025. https://www.chcs.org/media/CHCS-SDOH-Measures-Brief_120716_FINAL.pdf

[5] Gottlieb LM, DeSilvey SC, Fichtenberg C, Bernheim S, Peltz A. Developing national social care standards. Health Affairs Forefront. February 22, 2023. Accessed March 14, 2025. https://www.healthaffairs.org/content/forefront/developing-national-social-care-standards

[6] Samuels-Kalow ME, Boggs KM, Cash RE, . Screening for health-related social needs of emergency department patients. Ann Emerg Med. 2021;77(1):62-68. doi:10.1016/j.annemergmed.2020.08.010 33160720 PMC7755764

[7] Molina MF, Li CN, Manchanda EC, . Prevalence of emergency department social risk and social needs. West J Emerg Med. 2020;21(6):152-161. doi:10.5811/westjem.2020.7.47796 33207161 PMC7673900

[8] Samuels-Kalow ME, Mayes K, Cash RE, . Missed screening for adverse social determinants of health and emergency department utilization. Ann Emerg Med. 2024;83(4):416-418. doi:10.1016/j.annemergmed.2023.11.008 38142374 PMC10960706

[9] Levine DM, Linder JA, Landon BE. Characteristics of Americans with primary care and changes over time, 2002-2015. JAMA Intern Med. 2020;180(3):463-466. doi:10.1001/jamainternmed.2019.6282 31841583 PMC6990950

[10] Kellermann AL, Hsia RY, Yeh C, Morganti KG. Emergency care: then, now, and next. Health Aff (Millwood). 2013;32(12):2069-2074. doi:10.1377/hlthaff.2013.0683 24301388

[11] Malecha PW, Williams JH, Kunzler NM, Goldfrank LR, Alter HJ, Doran KM. Material needs of emergency department patients: a systematic review. Acad Emerg Med. 2018;25(3):330-359. doi:10.1111/acem.13370 29266523

[12] Eder M, Henninger M, Durbin S, . Screening and interventions for social risk factors: technical brief to support the US Preventive Services Task Force. JAMA. 2021;326(14):1416-1428. doi:10.1001/jama.2021.12825 34468710 PMC13460784

[13] Gottlieb L, Hessler D, Long D, Amaya A, Adler N. A randomized trial on screening for social determinants of health: the iScreen study. Pediatrics. 2014;134(6):e1611-e1618. doi:10.1542/peds.2014-1439 25367545

[14] Berkowitz SA, Hulberg AC, Hong C, . Addressing basic resource needs to improve primary care quality: a community collaboration programme. BMJ Qual Saf. 2016;25(3):164-172. doi:10.1136/bmjqs-2015-004521 26621916

[15] PRAPARE. Accessed February 25, 2025. https://prapare.org/

[16] RTI International. Accountable health communities (AHC) model evaluation: third evaluation report. November 2024. Accessed March 14, 2025. https://www.cms.gov/priorities/innovation/data-and-reports/2024/ahc-3rd-eval-report

[17] Rowe A, Knox M. The impact of the healthcare environment on patient experience in the emergency department: a systematic review to understand the implications for patient-centered design. HERD. 2023;16(2):310-329. doi:10.1177/19375867221137097 36541114 PMC10133779

[18] Furbacher J, Fockele C, Buono BD, . 2021 SAEM Consensus Conference Proceedings: research priorities for developing emergency department screening tools for social risks and needs. West J Emerg Med. 2022;23(6):817-822. doi:10.5811/westjem.2022.8.57271 36409957 PMC9683763

[19] Kraynov L, Quarles A, Kerrigan A, . Proceedings from the 2021 SAEM Consensus Conference: research priorities for interventions to address social risks and needs identified in emergency department patients. West J Emerg Med. 2023;24(2):295-301. doi:10.5811/westjem.2022.11.57293 36976612 PMC10047718

[20] Emergency Medicine Network. Massachusetts General Hospital, Harvard Medical School. Accessed September 7, 2024. https://www.emnet-usa.org/

[21] Sullivan AF, Richman IB, Ahn CJ, . A profile of US emergency departments in 2001. Ann Emerg Med. 2006;48(6):694-701. doi:10.1016/j.annemergmed.2006.08.020 17067721

[22] Herscovici DM, Boggs KM, Sullivan AF, Camargo CA Jr. What is a freestanding emergency department? definitions differ across major United States data sources. West J Emerg Med. 2020;21(3):660-664. doi:10.5811/westjem.2020.3.46001 32421516 PMC7234700

[23] Billioux A, Verlander K, Anthony S, Alley D. Standardized screening for health-related social needs in clinical settings: the accountable health communities screening tool. May 30, 2017. Accessed March 14, 2025. https://www.networktools.nchn.org/uploads/2/7/8/5/27858331/standardized-screening-for-health-related-social-needs-in-clinical-settings.pdf

[24] Kolenikov S. Calibrating survey data using iterative proportional fitting (raking). Stata J. 2014;14(1):22-59. doi:10.1177/1536867X1401400104

[25] Kanak MM, Stewart AM, Chang L, Fleegler EW. Health-related social risks versus needs in a pediatric emergency department. Am J Prev Med. 2024;67(2):291-295. doi:10.1016/j.amepre.2024.03.013 38555031

[26] Kushel MB, Gupta R, Gee L, Haas JS. Housing instability and food insecurity as barriers to health care among low-income Americans. J Gen Intern Med. 2006;21(1):71-77. doi:10.1111/j.1525-1497.2005.00278.x 16423128 PMC1484604

[27] Rodriguez RM, Fortman J, Chee C, Ng V, Poon D. Food, shelter and safety needs motivating homeless persons’ visits to an urban emergency department. Ann Emerg Med. 2009;53(5):598-602. doi:10.1016/j.annemergmed.2008.07.046 18838193

[28] Nagy J, Tedford N, Ahmed S, Thoms S, Kamimura A, Holsti M. Healthcare team members’ views on social determinants of health screening and referral practices in a pediatric emergency department. Patient Exp J. 2024;11(1):65-80. doi:10.35680/2372-0247.1877

[29] Hay S, Prabhala J, Umale N, . 156 Assessing perceptions of social determinants of health (SDoH) screening in the emergency department among patients and providers. Ann Emerg Med. 2023;82(4):S68. doi:10.1016/j.annemergmed.2023.08.178

[30] Children’s Hospital of Philadelphia. PolicyLab. Evaluating the impact of social risk screening on uptake of social assistance: leveraging research to ensure equity. Accessed November 14, 2024. https://www.policylab.chop.edu/project/socially-equitable-care-understanding-resource-engagement-secure-leveraging-research-ensure/printable/print

[31] Bouchelle Z, Vasan A. Promoting health equity through family-centered social needs screening and intervention in the inpatient setting. Hosp Pediatr. 2022;12(8):e275-e277. doi:10.1542/hpeds.2022-006725 35843956 PMC9390831

[32] Butler ED, Morgan AU, Kangovi S. Screening for unmet social needs: patient engagement or alienation? NEJM Catalyst. July 20, 2020. Accessed March 14, 2025. https://catalyst.nejm.org/doi/full/10.1056/CAT.19.1037

[33] Garg A, Boynton-Jarrett R, Dworkin PH. Avoiding the unintended consequences of screening for social determinants of health. JAMA. 2016;316(8):813-814. doi:10.1001/jama.2016.9282 27367226

[34] Seiber EE, Garrity K, Moon KJ, . Sustainability of social needs resolution interventions: a call to consider cost. Am J Prev Med. 2024;66(6):1100-1104. doi:10.1016/j.amepre.2024.01.010 38272244 PMC11102842

[35] Renaud J, McClellan SR, DePriest K, . Addressing health-related social needs via community resources: lessons from accountable health communities. Health Aff (Millwood). 2023;42(6):832-840. doi:10.1377/hlthaff.2022.01507 37196207

[36] Auerbach C, Mason SE. The value of the presence of social work in emergency departments. Soc Work Health Care. 2010;49(4):314-326. doi:10.1080/00981380903426772 20379902

[37] Bottino CJ, Rhodes ET, Kreatsoulas C, Cox JE, Fleegler EW. Food insecurity screening in pediatric primary care: can offering referrals help identify families in need? Acad Pediatr. 2017;17(5):497-503. doi:10.1016/j.acap.2016.10.006 28302365

[38] Schiavoni KH, Helscel K, Vogeli C, . Prevalence of social risk factors and social needs in a Medicaid Accountable Care Organization (ACO). BMC Health Serv Res. 2022;22(1):1375. doi:10.1186/s12913-022-08721-9 36403024 PMC9675191

[39] Mayes KD, Cash RE, Schiavoni KH, . Social risk, social need, and use of the emergency department. JAMA Netw Open. 2024;7(1):e2352365. doi:10.1001/jamanetworkopen.2023.52365 38241050 PMC10799261

