# Supplementary material for: Screening and Response for Adverse Social Determinants of Health in US Emergency Departments
Source: JAMA Netw Open. 2025 Apr 23;8(4):e257951. doi: 10.1001/jamanetworkopen.2025.7951 (PMC12019523; doi:10.1001/jamanetworkopen.2025.7951)
Supplement: Supplement 1. — eAppendix. Questions Regarding Emergency Department–Based Adverse Social Determinants of Health Screening Policies [file jamanetwopen-e257951-s001.pdf]

# Supplemental Online Content

Molina MF, Cash RE, Loo SS, et al. Screening and response for adverse social determinants of health in US emergency departments. *JAMA Netw Open*. 2025;8(4):e257951. doi:10.1001/jamanetworkopen.2025.7951

**eAppendix.** Questions Regarding Emergency Department–Based Adverse Social Determinants of Health Screening Policies

This supplemental material has been provided by the authors to give readers additional information about their work.

© 2025 Molina M et al. *JAMA Network Open*.

**eAppendix.** Questions Regarding Emergency Department–Based Adverse Social Determinants of Health Screening Policies

1) **What is the name of your hospital/ED?** ______________________________

2) **What is the mailing address of your hospital/ED?** ______________________________

3) **In 2022, was your ED open: a. 24 hours/day, 7 days/week?** ◯ YES ◯ NO **b. 365 days/year ?** ◯ YES ◯ NO

If **NO** to either question, please explain: ______________________________

4) **Please indicate the total number of patient visits at your ED and the 12-month reporting period to which they apply:**

# ED VISITS [ ][ ][ ][ ][ ][ ]

Reporting period: From ____/____ to ___/____
MM YY MM YY

5) **Please indicate the approximate number of ED visits by children (e.g., age <18 years)***

# CHILD ED VISITS [ ][ ][ ][ ][ ][ ]

Specify age cut-off if not <18 years: Age < ______years

6) For EDs that regularly treat adults: **Does your ED have a dedicated area for children only (e.g., dedicated beds)?***

◯ YES ◯ NO ◯ Not applicable (e.g., children's hospital)

7) **Do you have an identified coordinator(s) for pediatric emergency care in your ED?***

◯ YES
◯ NO

**7a. How many identified coordinators does your ED have?** ◯ 1 ◯ 2 ◯ ≥ 3

**7b. Please specify the type of coordinator(s):** *(check all that apply)*

☐ Physician coordinator(s) ☐ Nurse coordinator(s) ☐ Other (e.g., PA, admin):____________

**7c. How many total hours (weekly) do/does your identified coordinator(s) devote to the role?**

◯ <5 hours ◯ 5 - 9.99 hours ◯ 10 - 19.99 hours ◯ 20 - 39.99 hours ◯ ≥ 40 hours

8) **Does your ED *receive* telehealth services for patient evaluation from another facility in your health system or from an outside organization?***

◯ YES
◯ NO

**Does your ED utilize telehealth for:** *(check all that apply)*

☐ Pediatrics ☐ Psychiatry ☐ Dermatology ☐ Transfer coordination ☐ COVID-19
☐ Stroke/neuro ☐ Trauma ☐ Radiology ☐ Disaster preparedness ☐ Other:___________

9) **Does your hospital/ED *provide* telehealth services *out* for the evaluation of patients in other EDs?***

◯ YES ◯ NO ◯ Not sure

* additional info on the back of the booklet

10) **Is at least one attending physician (not resident) on duty in the ED 24 hours/day, 7 days/week?** *(Exclude on-call physicians).*

◯ YES
◯ NO

**If NO, when a physician is not on duty in the ED, is any physician available to the ED by two-way voice communication 24 hours/day, 7 days/week:**

10a. From within your hospital? ◯ YES ◯ NO ◯ Not applicable (e.g., freestanding ED)
10b. From outside of your hospital? ◯ YES ◯ NO ◯ Not applicable (e.g., freestanding ED)

11) **Of the following options, please select the one response that best describes the availability of Social Work Services in your ED:**

◯ ED-based Social Worker(s)
◯ Hospital Social Worker(s) based outside the ED
______________________________

**Are Social Work Services available in your ED 24 hours/day, 7 days/week?**

◯ YES ◯ NO

© 2025 Molina M et al. *JAMA Network Open*.

but can respond to the ED

- ◯ Mixture of ED-based and Hospital Social Worker(s)
- ◯ Other:
- ◯ None *(go to #12)*

© 2025 Molina M et al. *JAMA Network Open*.

12) **Please complete the table below to let us know if your ED has a <u>written policy</u> in place to screen for any of the listed social needs (a-h). The policy would include standardized triage questions or mandatory nurse assessments, but would <u>not</u> include questions asked at the provider's discretion.**
**If you answer "YES" to any (a-h), please also answer i and ii.**

| | | | | | i) Does your ED have a policy requiring <u>documentation</u> of the social need? | | ii) Does your ED have a policy requiring a <u>specified response</u> to the social need from clinicians or staff?† | |
|---|---|---|---|---|---|---|---|---|
| | | YES | NO | | YES | NO | YES | NO |
| a. | Housing instability or homelessness | ☐ | ☐ | If YES → | ☐ | ☐ | ☐ | ☐ |
| b. | Food insecurity or hunger | ☐ | ☐ | If YES → | ☐ | ☐ | ☐ | ☐ |
| c. | Difficulty obtaining transportation | ☐ | ☐ | If YES → | ☐ | ☐ | ☐ | ☐ |
| d. | Trouble paying for utilities | ☐ | ☐ | If YES → | ☐ | ☐ | ☐ | ☐ |
| e. | Intimate partner violence | ☐ | ☐ | If YES → | ☐ | ☐ | ☐ | ☐ |
| f. | Other exposure to violence | ☐ | ☐ | If YES → | ☐ | ☐ | ☐ | ☐ |
| g. | Substance use (e.g., alcohol, opioids) | ☐ | ☐ | If YES → | ☐ | ☐ | ☐ | ☐ |
| h. | Mental health (e.g., suicide, psychosis) | ☐ | ☐ | If YES → | ☐ | ☐ | ☐ | ☐ |

**If "NO" to all (a-h), the survey is complete.**

†**ii:** Recognizing that individual clinicians may activate additional resources at their discretion, we are specifically asking about ED policies and procedures here.

13) **If your ED requires any <u>documentation</u>:**

**13a. Who documents social needs?** *(check all that apply)*
- ☐ Registration staff
- ☐ Social worker/Care coordinator
- ☐ Clinical provider (RN/APP, resident, attending)
- ☐ Entered directly by patient/guardian
- ☐ Other: ________________________

**13b. How are social needs documented?** *(check all that apply)*
- ☐ Recorded in the electronic health record
- ☐ Recorded in local hospital database (separate from clinical health record)
- ☐ Other: ________________________

14) **If your ED has any policy requiring a <u>specified response</u>, what does that response include?** *(check all that apply)*
- ☐ Consultation (e.g., social workers)
- ☐ Provision of standardized information sheets
- ☐ Provision of individualized information (e.g., using Aunt Bertha, NowPow, UniteUs, or other individual resource mapping software)
- ☐ Other: ____________________________________________

____________________________________________

15) **Does your ED have any <u>written policy</u> regarding <u>follow-up</u> for patients with any identified social needs?**

◯ YES ◯ NO

© 2025 Molina M et al. *JAMA Network Open*.

**Thank you!**

If you have any questions, comments, or suggestions, please write them on the back of this form, or you may contact Dr. Margaret Samuels-Kalow at emnet@partners.org.

© 2025 Molina M et al. *JAMA Network Open*.
