# Supplementary material for: Screening and Response for Adverse Social Determinants of Health in US Emergency Departments
Source: JAMA Netw Open. 2025 Apr 23;8(4):e257951. doi: 10.1001/jamanetworkopen.2025.7951 (PMC12019523; doi:10.1001/jamanetworkopen.2025.7951)
Supplement: Supplement 2. — Data Sharing Statement [file jamanetwopen-e257951-s002.pdf]

Data Sharing Statement

Molina. Screening and Response for Adverse Social Determinants of Health in US Emergency Departments. *JAMA Netw Open*. Published April 23, 2025.
doi:10.1001/jamanetworkopen.2025.7951

Data
Data available: No

Additional Information
Explanation for why data not available: Data may be made available upon request to the Emergency Medicine Network (EMNet).
